# Trade Unionism in Mental Hospitals

**Published:** 1913-10-11

**Authors:** 


### TRADE UNIONISM IN MENTAL HOSPITALS.

### The Organisation of Asylum Workers.

The organisation of nurses and attendants and the formation of a feeling of comradeship beyond the limits of individual mental hospitals has been of slow growth. Though the Medico-Psychological Association was founded in 1841 as a bond of union between the medical officers of asylums throughout Great Britain and Ireland, and a nursing examination was established by them in 1890, thus bringing nurses and attendants into a certain relationship with this Association, the latter still remained the medical officers' society exclusively, with its own organ, *The Journal of Mental Science*, the contents of which, though frequently bearing upon asylum nursing and administration, remained a sealed book to the rank and file of employees.

Under these circumstances it occurred to the medical officers of the Northampton County Asylum (Drs. Green and Harding) and the superintendent of nurses there (Miss Laura Evans) that a useful purpose would be served by the establishment of a society to include the lower as well as the upper ranks of asylum service, with a journal which might be helpful in promoting the solidarity of the service and in placing before nurses and attendants particularly higher ideals of the work of ministering to the insane and of the attainments necessary to enable them worthily to perform their responsible duties.

### The Asylum Workers' Association.

At this juncture the valuable co-operation of the late Miss Honnor Morten, so well known in the nursing world, was secured, and owing mainly to the persevering efforts of the latter the Asylum Workers' Association came into existence in 1895. Its declared objects were: (1) to improve generally the status of asylum nurses and attendants, (2) to secure the sympathy and co-operation of all those interested in institutional work and efforts, and (3) to provide homes of rest for asylum workers debilitated in the course of their duties. Sir Benjamin Ward Richardson, the first president of the Association, spoke of its primary object being to secure the sympathy of the public with asylum workers in their little understood trials and worries; the second, to improve the attendants and nurses themselves as regards suitable training, the net result being the benefit of the patients, whose welfare was so much in the hands of those in immediate charge of them. In 1897 Dr. Shuttleworth, a retired asylum superintendent, became hon. secretary and still holds that office. An executive committee, representing all ranks in asylum service, having been elected, the Association gradually increased in numbers, its membership mounting up to 5,600 in 1911. The objects of its founders have been steadily kept in view under such presidents as Sir James Crichton-Browne, Sir John Batty Tuke, Sir W. J. Collins, and Sir John Jardine, the present holder of the office. At first there was admittedly some distrust of the new Association through a suspicion on the part of certain asylum authorities that it might savour of trade unionism; but this gradually disappeared, and at the present time some seventy superintendents are vice-presidents. To quote the words of Sir J. Crichton-Browne in one of his presidential addresses: "The Association has in it nothing of the nature of a trade union: it does not presume to dictate in the matter of wages, but it is calculated to give audible expression to the difficulties and hopes of all those engaged in asylum service, to inspire them with some little pride in their calling, to raise their professional status and value, and thereby to secure for them increased consideration and ultimately better remuneration."

### The Campaign for Pensions.

One of the earliest efforts of the Association was to secure, in the interests of the insane, greater permanency of service, and to attain this object the necessity of amelioration in some cases of the rates of pay for experienced workers, in others of diminished hours of duty and of increased creature comforts in the matters of food and of housing, were consistently pressed upon the notice of those in authority, including that of the Commissioners in Lunacy, who from the first showed themselves in sympathy with the objects of the Association. Above all, it was felt that in order to encourage attendants and nurses to regard asylum work as a permanent career a supreme effort must be made to secure for them the assured prospect of a definite pension, which they might claim as a right instead of an indulgence dependent upon the good will or caprice of a particular asylum committee. To this end local influence at Parliamentary elections was perseveringly organised for many years; and in the fulness of time, 1909, the then president, Sir William Collins, introduced as a private member's Bill, and carried successfully into law in a single session, the Asylum Officers' Superannuation Act, providing on a definite scale, at reasonable rates of contribution, retiring allowances for the staffs of public asylums throughout Great Britain and Ireland after prescribed periods of service. It is true that the Association had to cede certain contested points as to length of service in order to secure the passage of the Bill, but efforts are now in progress to obtain Parliamentary redress of certain residual grievances—*e.g.* the protracted period of service required to give claims to superannuation, especially in the case of women.

### Lancashire Attendants' Agitation.

It might have been thought that so large a concession to the aspirations of asylum workers, at first hailed with universal satisfaction, would have led to a period of comparative contentment. Agitation, however, soon sprung up amongst certain groups of attendants (notably in Lancashire), who having been exceptionally well treated by their asylum authorities prior to the passing of the Act, found that they had not scored thereby so much as their fellows in less-favoured districts. The outcome was the foundation in 1910 of a trade union, pure and simple, under the title of The National Asylum Workers' Union, which was registered (with headquarters at Manchester) under the Trade Union Acts in July 1911. Its objects are stated to be: "To obtain by legislation improvement of the status of asylum workers, reasonable hours of duty and of freedom, fair rates of wages, abolition of vexatious restrictions and other grievances, and a better regulation of relations between employers and employed; to assist members if wrongfully dismissed; to provide legal aid when necessary, and to promote the welfare of Asylum Workers generally."

### The Asylum Workers' Union.

Until recently its organising secretary has been the Rev. H. M. S. Bankart, B.A., ex-chaplain of the County Asylum at Lancaster. He also edited its monthly organ, the *N.A.-W.U. Magazine*, with considerable ability we are bound to admit, but, being sheltered by the ægis of the Trade Union Acts, with much vituperative energy and frequent "appeals to the gallery," calculated to arouse distrust and dissension amongst asylum employees, if not to disturb the needful discipline of a well-ordered institution, by representing the interests of the superior officers as opposed to those of the rank and file. An election pledge having been obtained from Lord Wolmer, M.P. for the Newton Division of Lancashire, a district containing a considerable number of asylum voters, his lordship brought forward in 1911, on behalf of the Union, a "Bill to limit the Hours of Employment of Officers and Servants in Asylums and to Amend the Asylum Officers' Superannuation Act, 1909." This was referred to a Select Committee, which reported in favour of the limitation of hours of the nursing staffs in asylums to seventy hours weekly (in lieu of sixty, as originally proposed). An amended Bill, embodying the chief recommendations of the Committee, which included the right of female officers and servants to claim a pension after twenty-five years' service, irrespective of age, was introduced by Lord Wolmer in the Session of 1913, but made no progress owing to the opposition of the Home Office and of the County Councils Association. It is stated that the Union has now between five and six thousand members.

### Attendants' other Organisations.

It is interesting to notice that the present Union is not the first that has been formed for asylum attendants. In 1896 a society entitled the National Union of Asylum Attendants in Ireland went so far as to issue rules for members, but premature publication roused the opposition of the asylum authorities, and the new bantling seems to have been strangled in its birth. In commenting on this event the *Journal of Mental Science* (July 1896) writes: "A trade union is as impossible in an asylum as in the Army or Navy. Discipline would be impossible, and no confidence could be placed on a staff which would at any moment be paralysed by the action of an irresponsible and often tyrannously autocratic trade-union committee." As has been well observed in a recent number of The Hospital (September 27) with reference to adumbrated strikes in asylums: "In any case a strike . . . could not be a 'down tools' in the accepted sense owing to the terms of service, which preclude any official from leaving without notice except at the sacrifice of a full month's wages and emoluments. Further, in the case of officials of over ten years standing 'a strike' would be a grave error, as they would sacrifice their right to the pension to which they contribute annually," and (as might have been added) the return of such contributions.

### The Need for Public Recognition.

In pointing out these considerations we do not impugn the abstract right of asylum workers to combine like any other body of workers to improve their position, and we freely admit that in some cases strong measures have been provoked by the economic tyranny of certain asylum authorities, and possibly (in a few instances) by the autocratic spirit of medical superintendents who have not kept abreast of the times. Yet, we would appeal to those asylum workers who take an enlightened view of their vocation to remember that theirs is a "high calling," and that the tending and nursing of the insane, exacting, as it does, the best qualities of heart and head, hardly falls under the same category as the daily job of the labourer, or even the mechanic. It is, in fact, a public service deserving liberal public recognition, but this may be obtained by other methods than the trade-union arbitrament of a strike.

